# Profile of natural *Trypanosoma cruzi* infection among dogs from rural areas of southern Espírito Santo, Brazil

**DOI:** 10.1590/0037-8682-0712-2021

**Published:** 2022-12-16

**Authors:** Beathriz Giostri Pontes, Marieta Cristina Couto Kuster, Letícia Azeredo de Freitas, Wagner Miranda Barbosa, George Luiz Lins Machado-Coelho, Marcos Santos Zanini, Maria Terezinha Bahia, Fabiane Matos dos Santos

**Affiliations:** 1 Universidade Federal do Espírito Santo, Programa de Pós-graduação em Ciências Veterinárias, Alegre, ES, Brasil.; 2 Universidade Federal do Espírito Santo, Departamento de Farmácia e Nutrição, Alegre, ES, Brasil.; 3 Universidade Federal de Ouro Preto, Escola de Medicina, Laboratório de Doenças Parasitárias, Ouro Preto, MG, Brasil.

**Keywords:** Chagas disease, Trypanosoma cruzi, Canis familiaris, Natural infection, Serology.

## Abstract

**Background:**

The emergence of *Trypanosoma cruzi* infection via oral transmission has a habitual character in its primitive endemic cycle. Recent findings revealed the first death by oral transmission of *T. cruzi* in the state of Espírito Santo, Brazil, in 2012, which was recorded in the rural area of Guarapari. This study evaluated the characteristics related to the occurrence of natural *T. cruzi* infection among dogs from the rural areas of Alegre and Iconha, municipalities of Espírito Santo.

**Methods:**

Logistic regression analysis of factors contributing to serological detection of *T. cruzi* in dogs was performed in environments where Espírito Santo’s Department of Health Surveillance had previously notified triatomines positive for *Trypanosoma* spp. from 2014 to 2017.

**Results:**

A total of 36 dogs were analyzed, of which 10 (27.77%) tested positive, one was borderline (2.79%), and 25 tested negative (69.44%) for *T. cruzi* infection. São Caetano, a district from the Iconha municipality, presented a 25 times greater chance for the detection of positive tests (OR:25; 95% CI; 2.37->100). Dogs with updated mandatory vaccination presented with a lower risk of positive serodiagnosis (OR:0.12; 95% CI: 0.02-0.63).

**Conclusions:**

Our results highlight for the first time the occurrence of natural *T. cruzi* canine infection, detected in the municipality of Iconha, mainly among dogs with un-updated mandatory vaccines in the district of São Caetano.

## INTRODUCTION

The protozoan parasite *Trypanosoma (Schyzotripanum) cruzi* (*T. cruzi*) is the etiologic agent of Chagas disease, an infectious disease classified by the World Health Organization as a tropical and neglected disease due to poor socioeconomic conditions of infected individuals living in endemic countries^1^ ^,^ ^2^. Characterized as the etiological agent of complex zoonosis, *T. cruzi* is mainly transmitted in the enzootic cycle by approximately 130 species of triatomines of the Reduviidae family, capable of infecting over 150 species of domestic and wild mammals^3^.

Brazil and other endemic countries in the southern cone of South America fortunately achieved a reduction in *T. cruzi* vectorial transmission after the development of a control program for *Triatoma infestans*, a domiciliated triatomine species^4^. However, oral *T. cruzi* transmission has become noteworthy in Brazil, mainly related to the incorrect manipulation and ingestion of food products from the vector ecosystem, or from direct contact with infected wild triatomines in endemic regions^5^ ^,^ ^6^. In this new epidemiological scenario of oral transmission, the Amazon region comprises approximately 92.3% human Chagas cases in Brazil, most of which are reported in Pará as a result of food contamination by infected triatomines, mainly due to improper handling of the açaí palm-tree fruit^7^.

The state of Espírito Santo, Brazil, has a low incidence of autochthonous human cases originating from infected sylvatic triatomine bugs in an epidemiological scenario wherein *Triatoma vitticeps* exhibits highest rate of natural infection, mainly attributed to its close relationship with wild mammals not refractory to *T. cruzi* ^8^ ^,^ ^9^. In 2012, a fatal acute case of 2-year-old was reported after accidental oral ingestion of one *T. cruzi*-infected triatomine in Rio da Prata, a rural area of Guarapari^6^. Therefore, some studies were conducted to understand the ecology of transmission cycles, considering environmental variables such as triatomine species and wild and domestic mammal species responsible for parasite maintenance surrounding Rio da Prata^6^ ^,^ ^10^. In this rural area of Espírito Santo state, *T. vitticeps* and *Panstrongylus geniculatus* were reported to be infected by *T. cruzi* along with wildlife species. In addition, domestic dogs play an important role as a link between the wild and domestic transmission cycles, as they have been shown seropositivity for *T. cruzi*. Dogs living in locations surrounding the 2012 death were considered a single event, regardless of the address of the acute Chagas death in Rio da Prata^6^ ^,^ ^10^.

Serological surveys performed in Northeast Brazil also found dogs naturally infected with *T. cruzi,* highlighting the role of these animals as parasite reservoirs in households, with the presence of vector *Triatoma brasiliensis* ^11^ ^,^ ^12^. Dogs parasitized by *T. cruzi* are considered sentinels for Chagas disease in enzootic areas^13^ ^,^ ^14^. Furthermore, the evolution of trypanosomiasis by *T. cruzi* in dogs is similar to the development of clinical forms found in chronic human cases, as demonstrated in experimental studies of pathologic and chemotherapeutic approaches using the dog model of *T. cruzi* infection^15^ ^,^ ^16^. 

A dog survey was conducted in the municipalities of Alegre and Iconha with the aim of evaluating the characteristics that support natural canine *T. cruzi* infection in an enzootic area with infected sylvatic triatomine bugs; both cities are in the south of Espírito Santo. 

## METHODS

### Geographic origin

Alegre is a municipality located in southeast Brazil, in southern Espírito Santo, covering a total area of 772.7 km^2^. The population was estimated to be 30,768 in 2010, which was Brazil’s last national census^17^. The climate is hot and rainy in the summer; however, dry in the winter, with an average annual temperature of 22.2°C, varying between 16.9 and 29.0°C. The landscape of Alegre is rugged and elevated, and shallow mineral soils can be found. The municipality is endowed with a vast and dense drainage basin, with the Atlantic Forest as its predominant biome and Rio Itapemirim as the main river. The local economy is based on agriculture.

Iconha is also located in southeastern Brazil, in southern Espírito Santo. Its municipality area is 202.9 km^2^ in total, and its population was estimated to be 12,523 in 2010^18^. The tropical climate has an average annual temperature of 23.0°C. The highest annual rainfall occurs between October and January. This municipality includes many mountains owing to its rugged topography, 15% wavy, and only 5% flat. Iconha confines itself to the west with Alfredo Chaves, a neighboring municipality of Guarapari. The local municipal economy is based on agriculture, especially banana production, and the urban area of Iconha has large networks of road carriers.

### Study area and sample definition

This cross-sectional study was conducted in the rural localities of Alegre and Iconha, Espírito Santo, Brazil. Each municipality was evaluated for existence of dogs living in households and peridomestic environments, with previous notification of triatomines positive for *Trypanosoma* spp. The evaluation considered several dog behaviors, clinical symptoms and signs commonly evaluated during an overall veterinary clinical anamnesis, even considering the classical limitation of signs that could be found, for example, in *T. cruzi*-infected asymptomatic animals or in the coexistence of other parasitic infections in animals naturally infected with *T. cruzi*.

The area included in this study was characterized by the presence of dogs in households and peridomestic environments, presenting triatomine bugs reported as positive for *Trypanosoma* spp. between 2014 and 2017, according to unpublished data provided by “Entomology and Malacology Center of Espírito Santo, State Health Department of Espírito Santo (Nemes-SESA/ES)”. 

Dogs living in regions with no notification of triatomines or with triatomines reported as negative for *Trypanosoma* spp. between 2014 and 2017 in the municipalities of Alegre and Iconha were excluded from the study. Another important exclusion criterion was non-consent of owners. Dogs that presented with aggressive behavior or other previously diagnosed diseases were also excluded.

### Ethical aspects

All procedures and experimental protocols were performed according to the CONCEA (National Council for Control of Animal Experimentation) and behavioral instructions for the use of animals in research from Brazil. Research project number 20/2018 was approved by the Research Ethics Committee on the Use of Animals of the Federal University of Espírito Santo (CEUA-UFES) on 09/04/2018. The owners of all the dogs included in this study signed an informed consent form that contained descriptions of all the procedures to be performed. The possibility of publication of results in scientific articles and disclosures at events, safeguarding the identity, as well as the freedom to suspend participation at any time, and the absence of monetary gratification were also described in the consent form.

### Dog survey

The active search for dogs was conducted with the support of community health agents in all domiciles and peri-domiciles previously characterized for inclusion in the study. In total, 27 domiciles and 27 peri-domiciles were researched, but only four households and 11 peri-domicile environments of *T. cruzi*-infected triatomine notifications effectively participated in the study during 2018 and 2019. Animal owners were interviewed regarding each dog’s demographic characteristics, habits, and clinical records. These characteristics were: age, sex, municipality of origin, district of origin, environment (domiciliary or peridomiciliary), area in the municipality (urban or rural), localization of dog (outside household or in transit), contactants (absent or people and animals), vaccination (updated or outdated), deworming (updated or outdated), ectoparasites (absence or presence), food - whether adequate (consisting of dog food) or inadequate (such as raw viscera from wild or domestic animals), Canine Body Mass Index-CBMI (eutrophy or irregular), Body Condition Score-BCS (eutrophy or irregular), whether normal or altered for hydration, capillary refill time, mucous, respiratory rate, heart rate, temperature, abdominal palpation, cardiopulmonary auscultation, lymph nodes (non-reactive or reactive), and normal or altered for any other clinical findings which included different signs detected at the moment of evaluation, such as secretions, excretions, discharge and volume increases. CBMI and BCS were measured to evaluate the clinical condition of the dogs^19^ ^,^ ^20^. 

Blood samples were collected by puncturing the cephalic vein, and after centrifugation, the serum samples were stored in microtubes and packed at -80 °C until enzyme-linked immunosorbent assay (ELISA) was performed. Blood samples were collected from each dog’s household following hygiene and safety standards. For the anti-*T. cruzi* immunoglobulin G (IgG) antibody assay using an in-house ELISA protocol, epimastigote forms of *T. cruzi* Y strain grown in acellular culture medium (liver tryptose infusion - LIT) were used as antigen^21^ ^,^ ^22^. The sera of 10 dogs maintained under experimental conditions at the Federal University of Ouro Preto were used as uninfected controls to estimate the cut-off through their average absorbance plus two standard deviations^23^ ^,^ ^24^. Furthermore, the sera of two other dogs experimentally infected with *T. cruzi,* also provided by the Federal University of Ouro Preto, were used as positive controls. Serum samples with absorbance values above the cut-off were indicative of reactivity for *T. cruzi* antigen, serum absorbance equal to at least one decimal case of the cut-off was classified as borderline, and values of absorbance below the cut-off were considered non-reagents. The ELISA was performed twice for all serum samples that showed borderline absorbance during the first examination. This in-house ELISA, previously validated for an experimental dog model of *T. cruzi* infection^16^ ^,^ ^23^ ^,^ ^24^, was used for the first time to test the serum reactivity for anti-*T. cruzi* immunoglobulin G (IgG) in dogs from rural endemic areas with parasitized reduviid bugs.

### Statistical analysis

All characteristic variables collected from each dog and the serology results of the dogs’ blood samples were used for statistical analysis. Serum samples classified as borderline and those with absorbance below the cut-off were grouped as non-positive. Binary logistic regression analysis was performed to estimate the probability of association or risk of detecting serological reactivity to *T. cruzi* antigens in the presence of other characteristic variables. Serological reactivity to *T. cruzi* antigens was chosen as the dependent variable, and dogs were categorized as either positive and non-positive, or reactive and non-reactive. Data were analyzed using the STATA version 14.1 (Stata Corp., College Station, United States), expressed as simple frequency and percentage, with 95% confidence interval. A significance level of 5% was used to evaluate the odds ratio (OR) for each variable.

## RESULTS

### Environmental evaluation and definition of dogs’ sample

**FIGURE 1:**
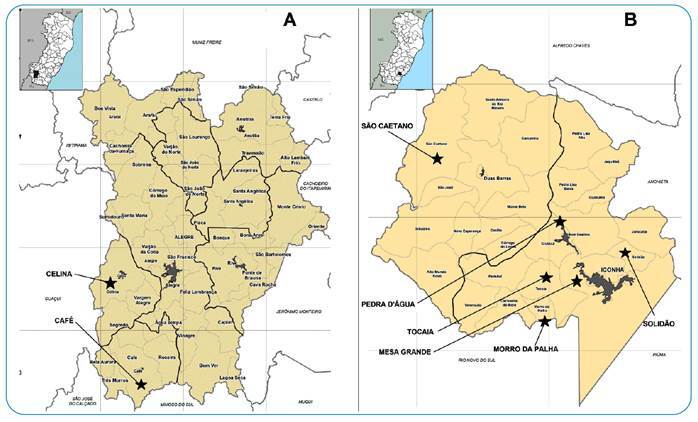
^25^ Representative maps (adaptation of IJSN, 2012)^25^ of the municipalities with dogs evaluated for *Trypanosoma cruzi* infection, due to the previous detection of triatomines positive for *Trypanosom*a *spp.* between 2014 and 2017: **(A)** Alegre, Espírito Santo state, Brazil; and **(B)** Iconha, Espírito Santo state, Brazil. The geographic area of localities with dogs evaluated in each municipality are highlighted.

**FIGURE 2:**
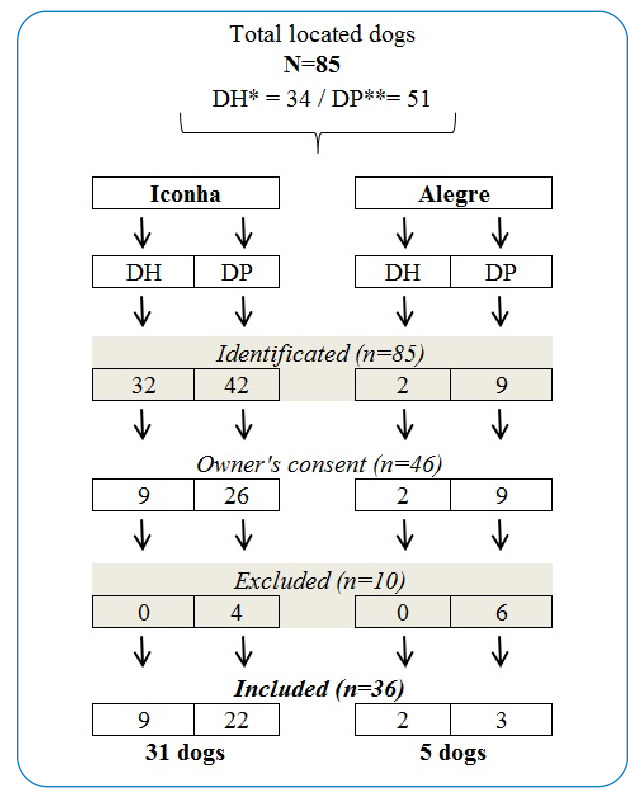
Flowchart of selected dogs in households and peridomiciles from regions notified with triatomines positive for *Trypanosoma spp.* between 2014 and 2017 in the municipalities of Alegre and Iconha, Espírito Santo, Brazil. *(DH) Dogs from households. **(DP) Dogs from peri-domiciles.

After exclusion, although other localities of Alegre and Iconha municipalities could have presented triatomines, a total of eight districts were evaluated in this study (Figure 1)^25^. In the municipality of Alegre (Figure 1A), the districts of Celina and Café were identified with two dogs living in households notified with *Trypanosoma* spp.-infected triatomines, and a total of nine dogs were detected in peridomiciles of places notified with triatomines positive for *Trypanosoma* spp. (Figure 2). In addition, six dogs from peridomestic environments were excluded because of their aggressive and inappropriate behavior. A total of five dogs were evaluated in Alegre, compriosing a sample of two dogs from domiciles and three dogs from peridomiciles notified with *Trypanosoma* spp*.-*infected triatomines between 2014 and 2017 (Figure 2). The triatomines previously reported by Nemes-SESA/ES were positive for *Trypanosoma* spp. in these localities from Alegre, which belonged to the *T. vitticeps* species.

Furthermore, six districts of Iconha were also integrated into the study region of study known by the following names: Solidão, Tocaia, Morro da Palha, Pedra D’Água, Mesa Grande, and São Caetano (Figure 1B). Firstly, 32 dogs from households and 42 dogs from peridomiciles were identified among these districts notified in Iconha with *Trypanosoma* spp.-infected triatomines between 2014 and 2017 (Figure 2). After the exclusion of dogs without their owners’ consent or exclusion of dogs with aggressive behavior, a total of 31 dogs were included in integrated the sample of animals assessed in the municipality of Iconha, nine dogs from households, and 22 dogs from peridomestic environments of notified places with triatomines positive for *Trypanosoma* spp. (Figure 2). Triatomines previously reported by Nemes-SESA/ES as positive for *Trypanosoma* spp. in these localities of Iconha between 2014 and 2017 belonged to *T. vitticeps* and *P. geniculatus* species.

### Risk factors for natural *T. cruzi* infection in dogs

**FIGURE 3:**
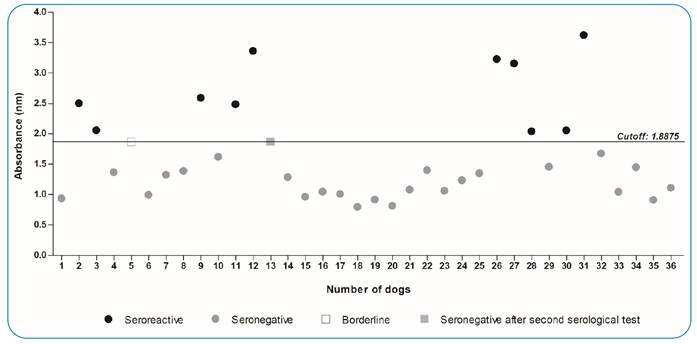
Serum level of anti-*Trypanosoma cruzi* antibodies detected in 36 dogs from households and peridomiciles previously reported with positive triatomines for *Trypanosoma spp.* in Alegre and Iconha municipalities, Espírito Santo state, Brazil.

**FIGURE 4:**
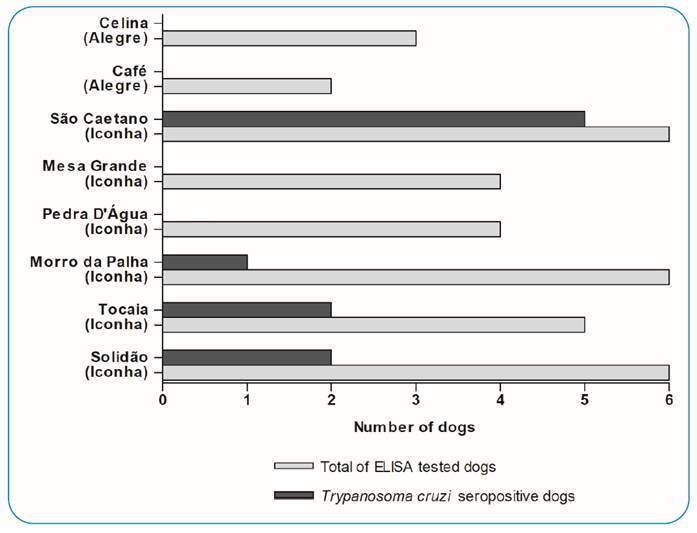
Locations evaluated in the municipalities of Alegre and Iconha, Espírito Santo state, Brazil, and quantitative identification of dogs that presented seropositive tests for detection of *Trypanosoma cruzi* antigens.

*T. cruzi* serological testing showed that 27.77% (10/36) of the assessed dogs were seropositive. One dog had a borderline anti-*T. cruzi* antigen, and the other 25 animals underwent a seronegative ELISA test (Figure 3). All *T. cruzi*-seropositive dogs lived in Iconha, two of which were from the district of Solidão, two dogs from Tocaia, and one animal from Morro da Palha, and 50% of seropositive dogs (5/10) lived in São Caetano (Figure 4). Although four dogs were evaluated in Pedra D’Água (Iconha-ES) and four dogs were also tested in Mesa Grande (Iconha-ES), none of them presented seropositive results for *T. cruzi* by ELISA (Figure 4). Additionally, five dogs were evaluated in Alegre, among which three dogs from the district of Celina and two from the district of Café tested seronegative for *T. cruzi* (Figure 4).

Concerning eco-epidemiological characteristics, a median age of 36 months was verified among the overall sample of dogs evaluated (95% CI = 5-168 months). Dogs that tested seropositive for *T. cruzi* had a median age of 36 months (95% CI = 18-72) similar to dogs with seronegative tests (median age, 95% CI = 5-168) (Table 1). The other characteristic variables analyzed were expressed as percentages of occurrence within the 36 dogs’ samples, followed by the percentages categorized as positive and non-positive dogs, since the serological reactivity to *T. cruzi* antigens was chosen as the dependent variable for bivariate logistic regression analysis (Table 1). Among all characteristic parameters measured, only district and vaccination variables were significantly associated with serological reactivity for *T. cruzi* (p < 0.05). Concerning the variable district, São Caetano district from Iconha municipality presented a 25 times greater chance of occurrence of *T. cruzi* seropositive dogs in relation to all the other seven districts evaluated in Alegre and Iconha municipalities (OR:25; p < 0.01). Furthermore, dogs with updated mandatory immunization protocols had an 88% lower risk of positive serological results for *T. cruzi* infection (OR:0.12; p < 0.05) (Table 1).

**TABLE 1:** Characteristic variables tested by binary logistic regression analysis to assess the existence of associations with serological reactivity to anti-*Trypanosoma cruzi* antibodies detected in 36 dogs from Alegre and Iconha municipalities, Espírito Santo state, Brazil.

Characteristic variables		Total		**Serological reactivity to anti-*Trypanosoma cruzi* IgG antibody**				Odds Ratio	Confidence interval (95%)		p value
				Seropositive		Seronegative					
Age (months)	36 (5-168)				36 (18-72)		36 (5-168)	1			
Sex (%)	*Female*	19	52.78%	5/19	26.31%	14/19	73.68%	1.66	0.27	5.02	0.836
	*Male*	17	47.22%	5/17	29.41%	12/17	70.58%				
Municipality (%)	*Alegre*	5	13.89%	0/5	0.00%	5/5	100.00%	1			
	*Iconha*	31	86.11%	10/31	32.25%	21/31	67.74%				
District (%)	*Other districts*	30	83.33%	5/30	16.66%	25/30	83.34%	25	2.37	262.65	0.007*
	*São Caetano*	6	16.67%	5/6	83.34%	1/6	16.66%				
Environment (%)	*Domiciliary*	11	30.56%	4/11	36.36%	7/11	63.63%	0.55	0.11	2.56	0.448
	*Peridomiciliary*	25	69.44%	6/25	24.00%	19/25	76.00%				
Area (%)	*Urban*	3	8.33%	0/3	0.00%	3/3	100.00%	1			
	*Rural*	33	91.67%	10/33	30.30%	23/33	69.69%				
Localization of dog (%)	*Outside*	19	52.78%	3/19	15.78%	16/19	84.21%	3.73	0.77	17.87	0.099
	*In transit*	17	47.22%	7/17	41.17%	10/17	58.82%				
Contactants (%)	*Absent*	0	0.00%	0/0	0.00%	0/0	0.00%	1			
	*People/animals*	36	100%	10/36	27.78%	26/36	72.22%				
Vaccination (%)	*Updated*	10	27.78%	6/10	60.00%	4/10	40.00%	0.12	0.02	0.63	0.012*
	*Outdated*	26	72.22%	4/26	15.38%	22/26	84.61%				
Deworming (%)	*Updated*	12	33.33%	4/12	33.33%	8/12	66.66%	0.66	0.14	3.03	0.60
	*Outdated*	24	66.67%	6/24	25%	18/24	75%				
Ectoparasites (%)	*Absence*	10	27.78%	4/10	40%	6/10	60%	0.45	0.94	2.14	0.316
	*Presence*	26	72.22%	6/26	23.07%	20/26	76.92%				
Feed (%)	*Adequate*	11	30.56%	2/11	18.18%	9/11	81.81%	2.11	0.36	12.15	0.4
	*Inadequate*	25	69.44%	8/25	32%	17/25	68%				
CBMI (%)	*Eutrophy*	11	30.56%	3/11	27.27%	8/11	81.81%	1.03	0.21	5.07	0.964
	*Irregular*	25	69.44%	7/25	28%	18/25	72%				
BCS (%)	*Eutrophy*	21	58.33%	8/21	38.09%	13/21	61.90%	0.25	0.44	1.4	0.116
	*Irregular*	15	41.67%	2/15	13.33%	13/15	86.66%				
Hydration (%)	*Normal*	35	97.22%	10/35	28.57%	25/35	71.42%	1			
	*Altered*	1	2.78%	0/1	0.00%	1/1	100.00%				
Capillary refill time (%)	*Normal*	35	97.22%	10/35	28.57%	25/35	71.43%	1			
	*Altered*	1	2.78%	0/1	0.00%	1/1	100.00%				
Mucous (%)	*Normocolored*	31	86.11%	9/31	29.03%	22/31	70.96%	0.61	0.05	62.464	0.678
	*Altered*	5	13.89%	1/5	20%	4/5	80%				
Respiratory rate (%)	*Normal*	11	30.56%	1/11	9.09%	10/11	90.90%	5.62	0.61	51.37	0.126
	*Altered*	25	69.44%	9/25	36%	16/25	64%				
Heart rate (%)	*Normal*	32	88.89%	9/32	28.12%	23/32	71.87%	0.85	0.77	9.30	0.895
	*Altered*	4	11.11%	1/4	25%	3/4	75%				
Temperature (%)	*Normal*	33	97.06%	10/33	30.30%	23/33	69.69%	1			
	*Altered*	1	2.94%	0/1	0.00%	1/1	100.00%				
Lymph nodes (%)	*Non-reactive*	31	86.11%	8/31	25.80%	23/31	74.19%	1.91	0.26	13.63	0.516
	*Reactive*	5	13.89%	2/5	40%	3/5	60%				
Abdominal palpation (%)	*Normal*	34	94.44%	10/34	29.41%	24/34	70.58%	1			
	*Altered*	2	5.56%	0/2	0.00%	2/2	100.00%				
Cardiopulmonary auscultation (%)	*Normal*	32	88.89%	10/32	31.25%	22/32	68.75%	1			
	*Altered*	4	11.11%	0/4	0.00%	4/4	100.00%				
Other clinical findings (%)	*Normal*	23	63.89%	8/23	34.78%	15/23	65.21%	0.34	0.06	1.93	0.22
	*Altered*	13	36.11%	2/13	15.38%	11/13	84.61%				

* Statistical significance (*p < 0.05*).

According to the binary logistic regression analysis, no significant associations were observed between serological reactivity for *T. cruzi* and all the other variables evaluated in this study (p > 0.05) (**Table 1**). However, it is noteworthy that these parameters were investigated for the first time in the study region, indicating interesting findings, mainly among 10 *T. cruzi* seroreactive dogs.

The current epidemiological profile of Chagas disease, mainly based on oral transmission, represents a new challenge for public authorities because previously employed control measures were not effective for this infectious disease, which is considered a foodborne illness. Some studies have shown the occurrence of natural *T. cruzi* infection in dogs, a sentinel reservoir for human infection, through vector and oral route transmissions in endemic areas^11^ ^-^ ^14^ ^,^ ^26^. The incidence of acute Chagas disease due to oral transmission is growing and expanding in several South American countries, including Brazil, mainly in the Amazon Basin^7^. Autochthonous human cases and acute oral transmission have also been reported in other localities characterized by wild triatomines branding the existence of *T. cruzi* biological cycle, as verified in Espírito Santo^6^. 

In the detection of anti-*T. cruzi* antibodies, our results demonstrated that dogs were exposed to infection by *T. cruzi* associated with non-domiciled bugs, mainly in the district of São Caetano in Iconha, which presented a 25 times greater chance of disease among the total number of animals evaluated in the other districts. Notably, our data showed the greatest risk of *T. cruzi* infection in dogs near Guarapari, in the region where death was verified by acute Chagas disease in 2012 and transmitted by oral ingestion of a wild *T. cruzi*-infected triatomine^6^. Previous results also indicated the presence of canine natural *T. cruzi* infection in Guarapari, varying from 8% to 13% of borderline seroreactivity and 28% of seroconversion to anti-*T. cruzi* antibodies in dogs near Rio da Prata^6^ ^,^ ^10^.

However, none of the five dogs from Alegre presented seroreactivity for anti-*T. cruzi* antibodies, although all of them lived in an area with non-domiciliated triatomines reported previously by Nemes-SESA/ES as infected by *Trypanosoma spp.* Other studies have shown the presence of the triatomine species *T. vitticeps* in 27 municipalities of Espírito Santo, including localities neighboring Alegre and Iconha. More than 85% of the triatomines captured were infected with flagellates morphologically similar to *T. cruzi* ^8^ ^,^ ^9^. Recently, molecular approaches confirmed the presence of the flagellate species *T. cruzi* infecting *T. vitticeps* and *P. geniculatus* from the Atlantic Forest in southeastern Espírito Santo, in regions such as Caparaó and Litoral Sul, which geographically encompass the municipalities of Alegre and Iconha^27^. Considering this background and the results of our study, future investigations using specific tools such as molecular approaches may be relevant to better clarify the role of dogs as sentinels of *T. cruzi* infection in the studied area surrounding Alegre, mainly because of the lower number of dogs evaluated in this municipality. 

The routine uses of domestic animals, mainly dogs, as sentinels to monitor the epidemiological risk of Chagas disease has been previously proposed, and the diverse spectrum of dog surveys performed in American countries has confirmed the risk for *T. cruzi* infection^11^ ^-^ ^14^ ^,^ ^26^ ^,^ ^28^ ^-^ ^30^. The seropositivity rate was 4.44% in the presence of anti-*T. cruzi* as demonstrated in dog sera from Sonora, Mexico, and the data highlighted the direct correlation between seropositivity rate in dogs and the presence of antibodies against *T. cruzi* in humans in different regions of Mexico^26^. Recent studies conducted in Brazil showed seropositivity levels of 11%^11^ and 40%^12^ for anti-*T. cruzi* antibodies among dogs from rural areas of Rio Grande do Norte state, and confirmed the relevant sentinel role of dogs as domestic reservoirs of *T. cruzi* in the biological cycle predominantly maintained by *Triatoma brasiliensis* in this endemic area^11^ ^,^ ^12^. 

In addition to the evaluation of the seropositivity rate of Chagas disease in dogs from regions with *T. cruzi*-infected triatomines, a broad analysis of findings in canine disease exceeds the elucidations regarding the development of control strategies to be implemented in endemic regions. Interesting epidemiological findings revealed that the habit of hunting and offering raw viscera and fresh blood to domestic dogs increased the risk of oral infection and transformed the dog into the main domestic reservoir for *T. cruzi* in the endemic Chagas disease region of a rural community in Chaco, Argentina^14^. This study evaluated diverse parameters measured in dogs from domiciles and peri-domiciles previously reported with *Trypanosoma* spp. infected triatomines in Alegre and Iconha, Espírito Santo, Brazil. Although only the district of São Caetano and updated vaccination were associated with the risk of canine natural Chagas disease, several other interesting parameters were measured for the first time in the geographical region studied, out of which the occurrence of inadequate feeding was verified in 32% of the dogs seropositive for anti-*T. cruzi* antibody.

Our results showed that dogs with updated mandatory vaccination protocols in this Brazilian region had a lower risk of seropositivity for *T. cruzi* infection. The mandatory vaccines for dogs in Brazil do not include a specific vaccine for canine Chagas disease, considering the non-availability of this vaccine to date. However, our data demonstrate for the first time in a natural endemic area that even a non-specific *T. cruzi* vaccine may offer a lower risk for natural canine *T. cruzi* infection. Perhaps future investigations will be able to better explain these results, probably implicated in the improvement of the innate immune system or in other responses to immunological benefits achieved by dogs with updated mandatory immunization protocols in the Brazilian municipalities of Alegre and Iconha. Research on vaccines to prevent *T. cruzi* infection has demonstrated the potential of finding effective prophylactic options^31^ ^-^ ^33^. Dogs immunized by a vaccine with non-pathogenic *Trypanosoma rangeli* epimastigotes had induced antibodies against *T. cruzi* for more than three years in their natural habitat in a rural area of Córdoba, Argentina^31^. The pBCSSP4 plasmid immunotherapy, a DNA vaccine treatment, preserved the cardiac structure and function to a greater extent, and prevented cardiomegaly in beagle dogs experimentally infected with *T. cruzi* ^32^. Recent findings of a vaccine established by a *T. cruzi* strain expressing GFP-DDDHA induced by trimethoprim-lactate, which results in the death of intracellular parasites, revealed a very strong protection against re-infection in mice^33^. Immune cells demonstrated earlier and stronger protective responses in immunized mice after re-infection with *T. cruzi* ^33^.

## CONCLUSIONS

An overall analysis of our findings implies efforts to search for control strategies and prophylactic options to prevent *T. cruzi*-infectivity spread by non-domestic triatomines that have access to human domiciles in enzootic rural areas, since dogs with updated mandatory vaccines presented a lower risk of natural infection in this endemic scenario. To the best of our knowledge, this is the first time that epidemiological factors involving *T. cruzi* natural infection with the participation of dogs as sentinels of the disease have been analyzed in the southern Espírito Santo Brazilian municipalities of Iconha and Alegre. The greatest risk for *T. cruzi* infection was verified among dogs from São Caetano, the nearest rural district to the previous notification of fatal acute oral Chagas disease in Espírito Santo. Future studies should evaluate the efficacy of specific dog vaccines to enhance the level of human protection by decreasing *T. cruzi* parasite load in triatomines and hosts dispersed in this new scenario of growing risk to oral transmission and human infectiousness in the endemic regions of Chagas disease.
